# *In vivo* recordings in freely behaving mice using independent silicon probes targeting multiple brain regions

**DOI:** 10.3389/fncir.2023.1293620

**Published:** 2023-12-22

**Authors:** Emanuel Ferreira-Fernandes, Mariana Laranjo, Tiago Reis, Bárbara Canijo, Pedro A. Ferreira, Pedro Martins, João Vilarinho, Mahmoud Tavakoli, Carolina Kunicki, João Peça

**Affiliations:** ^1^CNC - Center for Neuroscience and Cell Biology, University of Coimbra, Coimbra, Portugal; ^2^Institute of Interdisciplinary Research (IIIUC), University of Coimbra, Coimbra, Portugal; ^3^PhD Program in Experimental Biology and Biomedicine (PDBEB), University of Coimbra, Coimbra, Portugal; ^4^Department of Architecture, University of Coimbra, Coimbra, Portugal; ^5^Institute of Systems and Robotics, Department of Electrical and Computer Engineering, University of Coimbra, Coimbra, Portugal; ^6^Vasco da Gama Research Center (CIVG), Vasco da Gama University School (EUVG), Coimbra, Portugal; ^7^Department of Life Sciences, University of Coimbra, Coimbra, Portugal

**Keywords:** *in vivo* electrophysiology, silicon probes, methodology, mouse model, prefrontal cortex, hippocampus

## Abstract

*In vivo* recordings in freely behaving animals are crucial to understand the neuronal circuit basis of behavior. Although current multi-channel silicon probes provide unparalleled sampling density, the study of interacting neuronal populations requires the implantation of multiple probes across different regions of the brain. Ideally, these probes should be independently adjustable, to maximize the yield, and recoverable, to mitigate costs. In this work, we describe the implementation of a miniaturized 3D-printed headgear system for chronic *in vivo* recordings in mice using independently movable silicon probes targeting multiple brain regions. We successfully demonstrated the performance of the headgear by simultaneously recording the neuronal activity in the prelimbic cortex and dorsal hippocampus. The system proved to be sturdy, ensuring high-quality stable recordings and permitted reuse of the silicon probes, with no observable interference in mouse innate behaviors.

## Introduction

Electrophysiological recordings in freely behaving animals are critical to investigate neuronal activity during more naturalistic behaviors, providing real-time access to both extracellular action potentials and local field potentials (LFP) (Adrian and Moruzzi, 1939; Vanderwolf, 1969; O’Keefe and Dostrovsky, 1971). Despite the emergence of highly promising methodologies to monitor neuronal activity, particularly voltage-sensitive dye imaging techniques (Grinvald and Hildesheim, 2004; Newton et al., 2021), electrophysiological recordings retain their value as a research tool, due to their unparalleled temporal and spatial resolution and because their biophysical principles are well understood (Buzsáki et al., 2012). Current multi-site electrophysiological recordings are mostly based on tetrodes and silicon probes (McNaughton et al., 1983; Gray et al., 1995; Csicsvari et al., 2003; Buzsáki, 2004; Blanche et al., 2005; Jun et al., 2017). Tetrodes are a popular solution as they have cost benefits and can be independently positioned and adjusted to target virtually any combination of brain structures, despite the considerable manual labor required to assemble the brain implants (Wilson and McNaughton, 1993; Gray et al., 1995). Silicon probes, on the other hand, are more expensive and lack the targeting flexibility of tetrodes, but do not require assembly, cause minimal tissue displacement (Buzsáki, 2004; Kipke et al., 2008), their geometry can be customized to better suit the architecture of the brain structure of interest (Wise and Najafi, 1991), and new integrated designs are available, which, for instance, combine silicon probes with μLEDs for optogenetics (Wu et al., 2015; Kim et al., 2020). Therefore, silicon probes have become an increasingly attractive solution, driving the development of in-house implantation techniques to mitigate their limitations in terms of cost and lower targeting flexibility.

An ideal headgear should guarantee stable, long-term recordings, be printable and modular, accommodate multiple silicon probes, allow their independent movement and recovery for reimplantation, and should not interfere with animals’ behaviors. Such a headgear will provide cost-effective, high-quality data from multiple brain structures simultaneously, maximizing productivity, and minimizing the discomfort for the animals during surgery without disrupting its free behaviors. In contrast with the progress in recording electrodes, implantation techniques have lagged behind, particularly for silicon probe implantation. In fact, advanced implantation techniques such as the SLIQ drive (Liang et al., 2017; Ferreira-Fernandes et al., 2019), Flexdrive (Voigts et al., 2013), Shuttledrive (Voigts et al., 2020), DMCdrive (Kim et al., 2020), Hyperdrive (Lu et al., 2018), and TetrOdrive (Brosch et al., 2021), among other solutions (Sun et al., 2022), are only available for multi-site recordings with tetrodes. For silicon probe users, companies provide commercial protocols where the silicon probes are irreversibly attached to the skull, directly or through a microdrive to move the electrode after surgery, and the implants are covered with cement. To fill this gap, a few advanced headgears for silicon probes have emerged, either using disposable 3-D printed customized components (Headley et al., 2015; Chung et al., 2017; Guardamagna et al., 2022), mostly focused on reducing costs and manual labor; or reusable 3-D printed metal parts (Vöröslakos et al., 2021), which facilitate the recovery of silicon probes and other portions of the headgear. While these headgears are still restricted to a few laboratories, they provided a valuable turning point, motivating silicon probe users to learn from and adapt these new solutions to their own experimental needs.

Here, we report our implementation of a 3-D printed headgear system, based on the 3-D printed headgear developed by Vöröslakos et al. (2021) which allows chronic *in vivo* recordings in freely behaving mice using independently movable silicon probes targeting multiple brain regions. This headgear and associated protocols were tested for mouse electrophysiology, ensure the simultaneous and independent targeting of, at least, 2 brain structures, and allow the reliable recovery and reuse of the silicon probes and commercial, high precision nanodrives, decreasing costs, and experimenter effort, while causing no observable interference in mouse behaviors.

## Results

### The headgear system for chronic multi-site recordings in freely behaving mice

**Figure 1:**
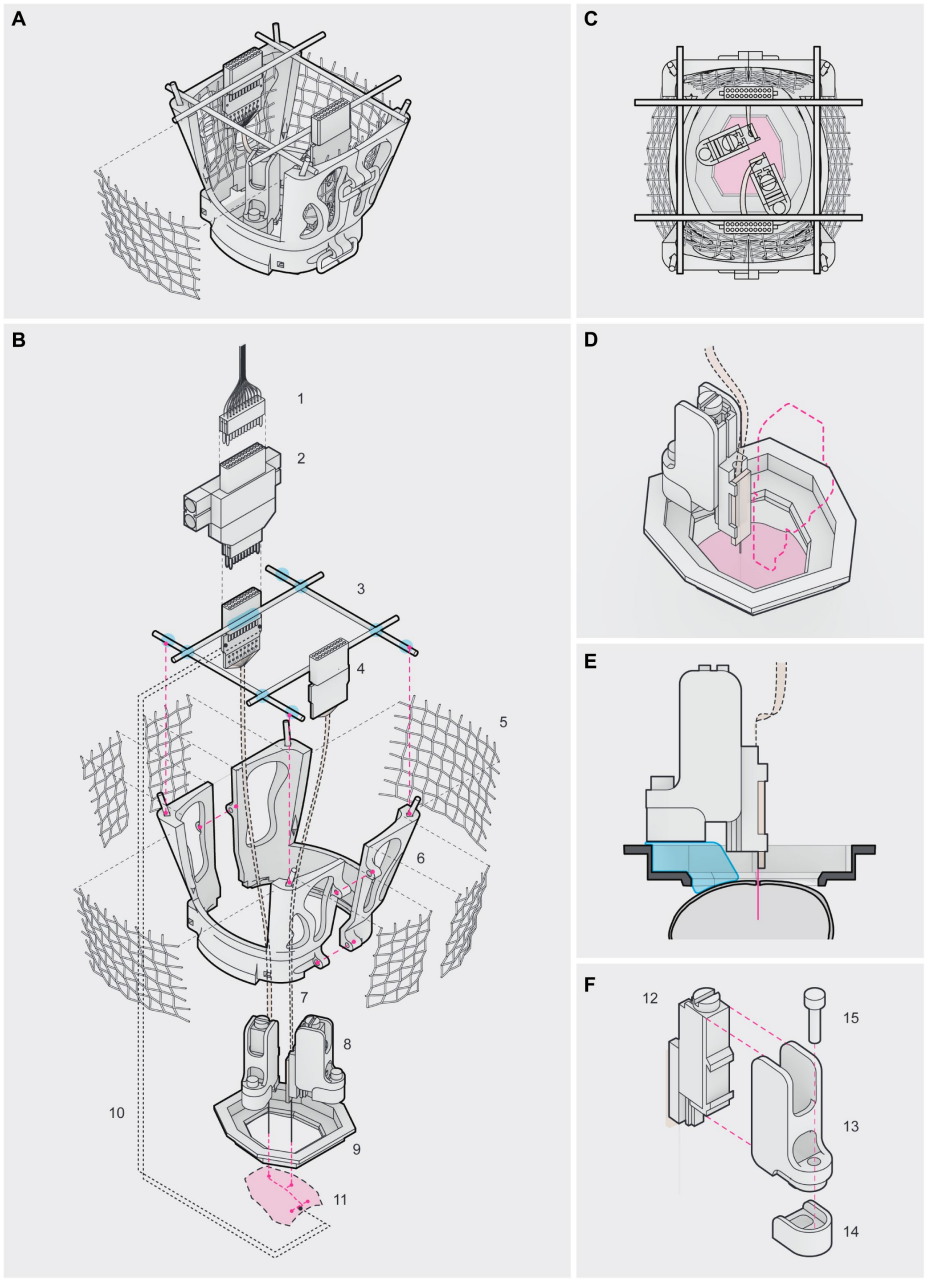
Headgear for chronic multi-site recordings in freely behaving mice **(A–C)**. Lateral **(A)**, top **(B)** and exploded **(C)** views of the fully assembled headgear. (1) ultra-fine cable, connecting the headstage to the Omniplex Neural Recording Data Acquisition System; (2) headstage; (3) male header pins (blue marks depict soldering/gluing points); (4) Omnetics connector; (5) aluminum mesh; (6) lateral wall; (7) flex cable; (8) nanodrive in its encasement; (9) circular base; (10) common ground wire; (11) surface of the skull (red marks depict the PL craniotomy at the front, the dCA1 craniotomy in the middle and the 2 posterior insertion points for the stainless screws). Note in **(A)** the 2 posterior wires used to close the cap. **(D–F)** Top **(D)**, lateral **(E)** and exploded **(F)** views of the nanodrive and its encasement. (12) metal nanodrive; (13) encasement’s frame; (14) detachable base and (15) stainless screw. To ensure silicon probe reuse, the nanodrive encasement was secured to the circular base and skull using self-adhesive resin (blue mark) in **(E)** applied onto the detachable base. Note that our circular base was flattened **(E,F)**. Once the recording experiments were finished, the silicon probe, the nanodrive and the encasement’s frame were recovered by removing the screw connecting the frame to the detachable base **(F)**. Note the elevated border and the groove in the detachable base designed to stabilize the encasement against torque, during the adjustments **(F)**.

In this work, we implemented a modular headgear system based on Vöröslakos and colleagues (Vöröslakos et al., 2021) with modifications, which combined 3D printable parts and commercial elements, to perform chronic *in vivo* recordings in freely behaving mice with independently movable silicon probes targeting multiple brain regions. Once assembled, the headgear consisted of a protective cap, 2 nanodrives and their encasements, 2 Omnetics connectors, 2 stainless screws, 2 common ground wires, and 2 silicon probes, which simultaneously targeted dorsal CA1 (dCA1) and the prelimbic (PL) cortex (Figures 1A-C). The protective cap was composed of a circular base and 2 lateral walls (Figure 1C). The circular base was directly attached to the skull of the animal using self-adhesive resin, which served as the main support for the cap. The internal window of the circular base was shaped as an elongated octagon, to match the outer ridge of the skull and ensure access to a wide range of brain regions. In our protocol, we flattened the circular base (modification #1) to better anchor the nanodrives, as shown in Figure 1D. The lateral walls were attached to the circular base using a rail and 3 wires and provided both structural support and protection to the silicon probes and electronics (Figure 1C).

The metal nanodrives were acquired from Cambridge Neurotech (modification #2). To ensure the reuse of the silicon probes, we designed an encasement to accommodate the commercial nanodrives (modification #3), consisting of a 3D printed frame and a detachable base connected by a stainless screw (Figures 1E,F), following the concept introduced by Vöröslakos et al. (2021). We decreased the thickness (to 1 mm) and bulkiness of the detachable base to minimize its impact on the dynamic range of the silicon probes and save space inside the protective cap (Figure 1F). The elevated border and a groove in the detachable base stabilized the encasement against torque during the adjustments (Figure 1F). Once a nanodrive fitted inside the frame, the detachable base allowed recovery of the nanodrives and silicon probes between surgeries, since only the detachable base was irreversibly secured to the skull with self-adhesive resin (Figures 1E,F). The commercial nanodrives used had a total travel distance of 5 mm (210 μm / turn) and came with a stereotaxic holder.

This headgear system weighed on average 5.440 ± 1.011 g per animal, evaluated by subtracting the pre-surgical weight of the animal to its post-surgical weight. The contribution of the different headgear parts was the following: circular base: 0.2835 ± 0.0042 g; lateral walls with male header pins and aluminum mesh: 0.8334 ± 0.0166 g, each; nanodrive and encasement: 0.4316 ± 0.0054 g, each; screws: 0.02422 ± 0.0004 g, each; Omnetics connectors: 0.3371 ± 0.0001 g, each. The remaining difference corresponded to the self-adhesive resin and other minor elements (e.g., the copper wires). A chronically implanted mouse could carry the headgear for weeks to months (up to 5 months), without visible deterioration of the implant. In our hands, the number of successful probe recoveries varied from 2 (initial attempts) to 6 per individual probe (see Supplementary Table S1, mice #1 to #6). The recordings reported in this work were carried out on 6 double implanted mice (see Supplementary Table S1, mice #1 to #6), using only a total of 3 silicon probes (see Supplementary Table S1, probe 1 to 3). Altogether, this modular headgear is sturdy, easy to assemble, and allows effective recovery and reuse of the silicon probes.

### Implanted animals did not show observable behavioral abnormalities

**Figure 2:**
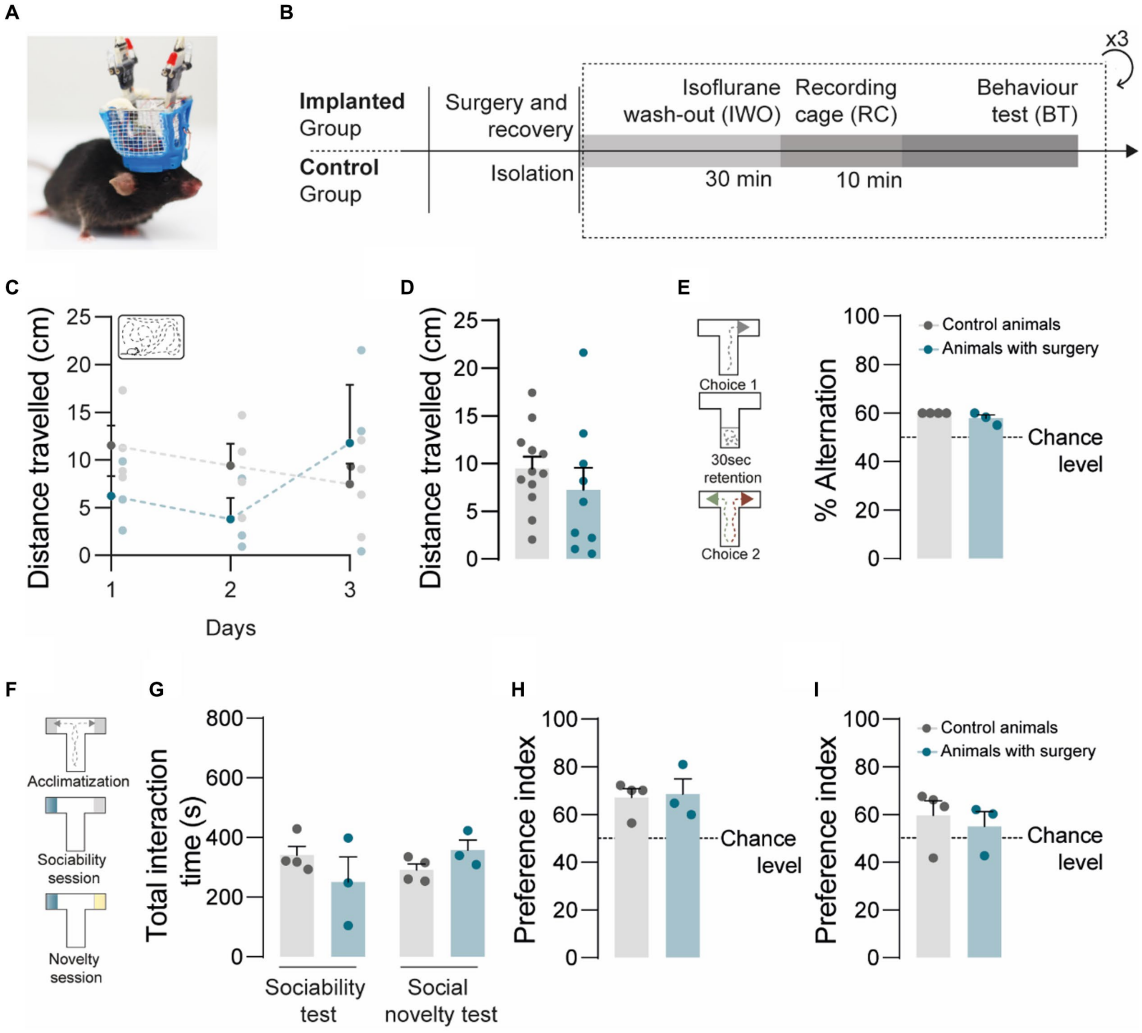
Implanted animals show no major abnormalities in social and non-social behaviors. **(A)** Example of an implanted mouse with headgear attached to the preamplifier and recording cable. **(B)** Experimental timeline for the implanted and control groups. **(C,D)** Implanted animals and controls travelled comparable distances in the recording cage, in the 3 consecutive days of testing **(C)** and when trials were averaged per animal **(D)**; two-way ANOVA, *F*(2,15) = 1.812, *p* = 0.1973 for **(C)** and Kolmogorov–Smirnov test; *p* = 0.6571 for **(D)**. **(E)** Both groups of animals alternated above chance level (50%), in the spontaneous alternation T-maze test; Kolmogorov–Smirnov test; *p* = 0.1429 between control animals and animals with surgery, Wilcoxon signed-rank test against the hypothetical value of chance alternation at 50%. **(F)** Schematic representation of the social T-maze test: acclimatization epoch, for habituation; ‘social/empty’ epoch, for sociability; and ‘familiar/novel’ epoch, for social novelty preference. **(G)** In the social T-maze, the total interaction time is similar in both groups and in both test sessions; Kolmogorov–Smirnov test; *p* = 0.4000. **(H)** In the sociability part of the test, both groups displayed social preference, spending more time in the social chamber compared to the empty chamber; Kolmogorov–Smirnov test; *p* = 0.8857 between control animals and animals with surgery, Wilcoxon signed-rank test against the hypothetical value of chance alternation at 50%. **(I)** In the novelty session, both groups tended to spend more time with the novel animal when compared to the familiar animal; Kolmogorov–Smirnov test; *p* = 0.2286 between control animals and animals with surgery, Wilcoxon signed-rank test against the hypothetical value of chance alternation at 50%. Control animals *n* = 4; Animals with surgery *n* = 3. All data are presented as means ± s.e.m. Statistical significance: *p < 0.05, **p < 0.01 and ***p < 0.001.

The implantation of optoelectronic devices in small rodents have the potential to interfere with animal behavior under laboratory settings (Yamamoto and Wilson, 2008; Vöröslakos et al., 2021). To assess the behavioral impact of the headgear, we compared the performance of implanted and non-implanted mice (Figure 2A) in 2 behavioral paradigms, the spontaneous alternation T-maze test (Edfawy et al., 2019) and the social T-maze test (Figure 2B). We started by evaluating behavior in the recording cage for 10 min, in 3 consecutive days, and estimated the daily distance traveled (Figures 2C,D) by each of the animals. We found no significant differences both in the daily distance (Figure 2C) and in the average distance (Figure 2D) traveled by implanted and non-implanted mice, suggesting that the headgear did not impose additional constraints and did not impact the quantitative aspects of rodent spontaneous movement. Next, we asked whether implanted animals would develop spatial biases, for which we compared the performance of implanted and non-implanted mice in the spontaneous alternation T-maze test. The experimental groups did not show significant differences in the percentage of alternation (Figure 2E), compatible with the absence of spatial biases putatively associated to tension in the cables or to the asymmetric distribution of the extra weight imposed by the components of the headgear onto the skull. While the headgear had no apparent impact in rodent spatial navigation and general motion, it might be disruptive for behavioral paradigms requiring social behavior, by acting as a stressful stimulus, a foreign object, or a distractor. To address this, we compared the social behavior of implanted and non-implanted mice undergoing the social T-maze test, which is our adapted version of the 3-Chamber test (Rein et al., 2020). In our protocol, the experimental animal is allowed to interact with 2 stimulus animals enclosed in the social chambers, reducing the exploration of unnecessary parts of the arena and imposing a discrete choice point, which simplifies data analysis and behavior annotation. For the social T-maze test, each experimental animal underwent 3 consecutive 10-min epochs per day: the acclimatization epoch, for habituation; the ‘social/empty’ epoch, to test for social preference; and the ‘familiar/novel’ epoch, to test for social novelty preference (Figure 2F). To characterize social behavior, we evaluated the total interaction time in ‘social/empty’ epochs and ‘familiar/novel’ epochs (Figure 2G), the percentage of time spent with the social stimulus in ‘social/empty’ epochs (Figure 2H), and the percentage of time spent with the novel mouse in ‘familiar/novel’ epochs (Figure 2I). In the ‘social/empty’ epochs, the experimental groups did not significantly differ in the total interaction time (Figure 2G), and both groups displayed a social preference index above chance and without significant differences (Figure 2H), suggestive of similar levels of sociability between implanted and non-implanted mice. Social interactions were not hindered by the plexiglass barriers, since we explicitly observed both reciprocal and non-reciprocal interactions during behavior annotation, including nose-to-nose contacts, sniffing, unilateral starring, and bilateral starring. The maintenance of sociability levels was further corroborated in ‘familiar/novel’ epochs, since we found no significant differences in the total interaction time between experimental groups and in comparison, to the ‘social/empty’ epochs (Figure 2H). Regarding social novelty preference, implanted and non-implanted mice did not show significant differences in the novelty preference index (Figure 2I). Our data on social novelty preference also suggests the existence of both novelty-seeking individuals and social neophobic individuals in our sample (Figure 2H). Together, these observations validated the social T-maze test as an alternative to the 3-Chamber test to probe sociability and social novelty preference in mice. While our available data suggests our headgear has no gross impact on behavior, future work will be required to determine the presence of subtle changes in discrete paradigms.

### Representative unit analysis in the PL cortex

**Figure 3:**
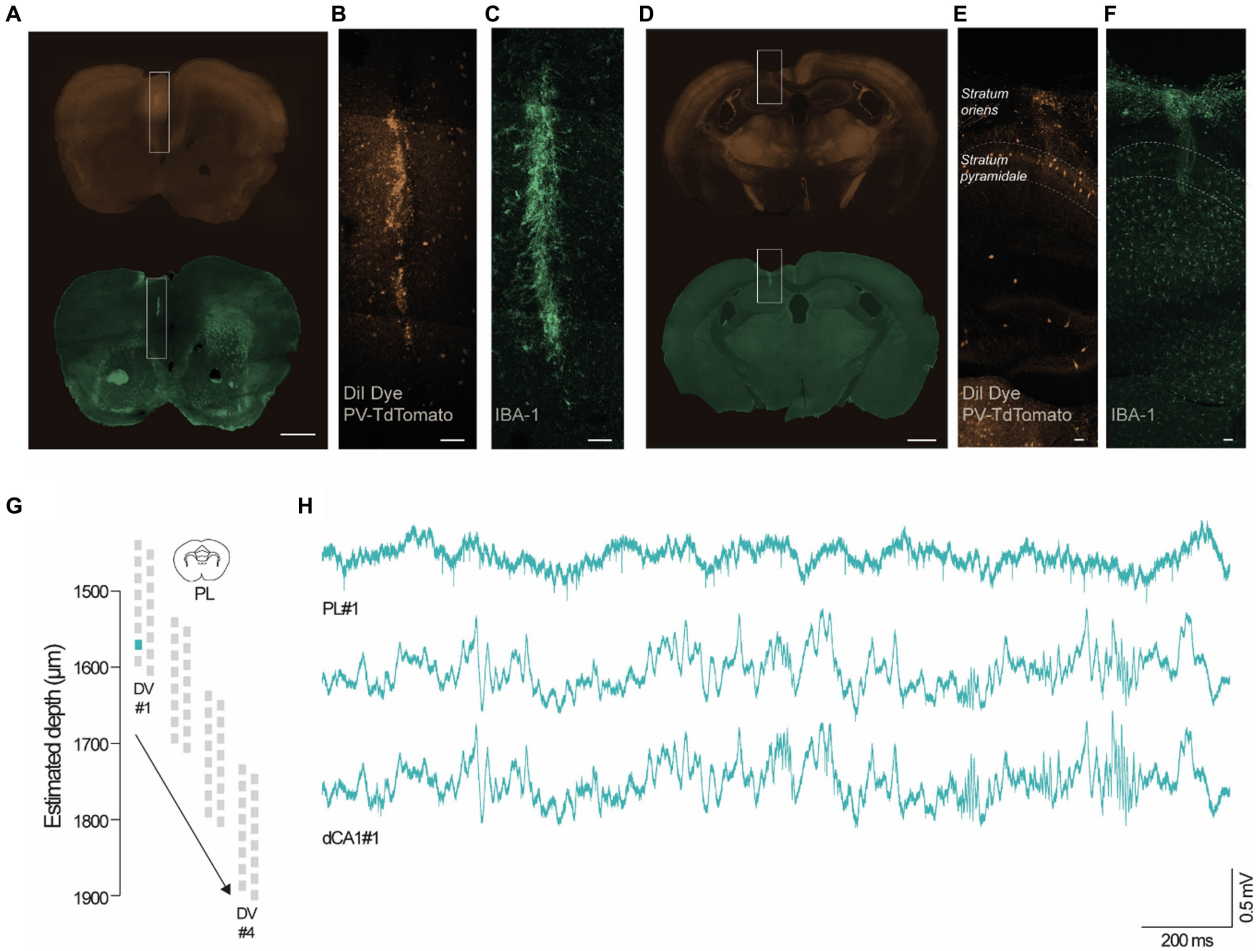
Representative dual site recordings in the PL cortex and dCA1. **(A–F)** Coronal sections of adult mice (4–5 months) showing the endogenous TdTomato staining in PV-positive neurons **(B,E)** and IBA-1 staining in microglia **(C,F)**. **(A,D)** The inserts highlight the location of the silicon probes in the PL cortex and dCA1 (scale bars, 50 μm). The probe tracks were identified using DiI **(B,E)** and IBA-1 reactivity **(C,F)** (scale bars, 50 μm). **(G,H)** Representative wide-band traces from adult mice recorded in the PL cortex and dCA1 using 2 16-channel silicon probes at DV#1 (PL, DV: 1600 μm; dCA1, DV:1150 μm) during the social T-maze test.

**Figure 4:**
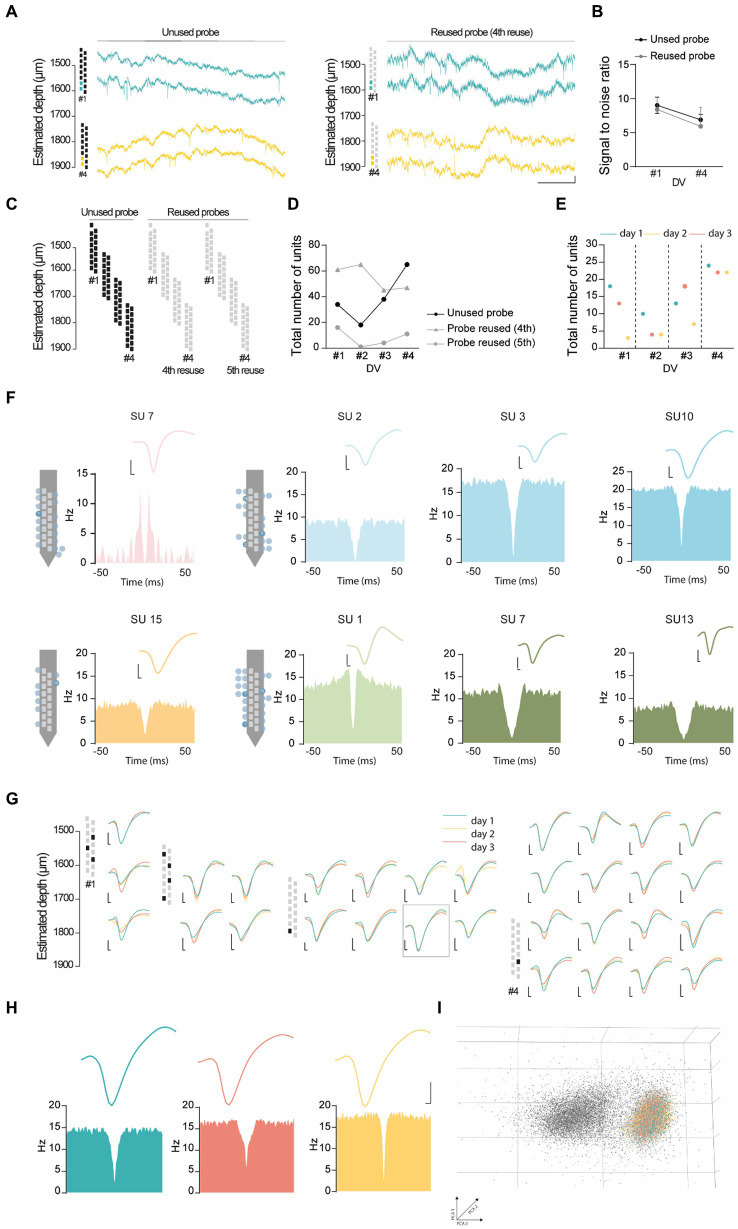
Representative unit analysis in the PL cortex. **(A)** Representative raw wide-band data from adult mice recorded in the PL cortex using 2 different 16-channel silicon probes – a unused probe (left) and a probe in its 4th reuse (right), in the recording cage at post-op day 7 (DV#1) and 22 (DV#4). **(B)** Signal to noise ratio computed from the probes used in **(A)**, at DV#1 and DV#4. **(C,D)** Total number of units (SUs + MUs) recorded across 3 consecutive days per DV (#1 to #4), using two different 16-channel silicon probes - an unused probe and a probe in its 4th and 5th reuse. **(E)** Total number of units (SUs + MUs) recorded using an unused probe. The recordings were performed across 3 consecutive days (day 1 to day 3) for each recording depth (#1 to #4). **(F)** Single unit diversity recorded in different depths with average waveforms and auto-correlograms. **(G-I)** SUs stability in 3 days of recordings in the same DV from the unused probe. **(G)** Average waveforms and ACGs of stable single units across multiple days, color-coded by recording day. **(H)** Putative stable single unit recorded across 3 days in the PL cortex, color-coded by recording day. **(I)** Representative PCA of the single unit depicted in **(H)**. The gray cluster in the background corresponds to the spikes of other units recorded during the same period as the single unit of interest. Scale bars in **(F-H)**: x-axis, 25 ms, y-axis, 0.05 mV.

To evaluate the performance of the headgear, we carried out dual-site recordings in the PL cortex (Figures 3A–C) and dCA1 (Figures 3D–F) during 10 min-epochs in the recording cage. To confirm the location of the recording sites, we coated the silicon probes with DiI (Nitzan et al., 2020) (Figures 3B,E), a lipophilic dye commonly used for histological identification of the probe tracks. Despite its effectiveness, DiI application required touching or dipping the silicon probes, thus increasing the probability of damaging the equipment. Since chronic recordings cause the activation of glial cells around the probes (Kozai et al., 2012), we used an alternative protocol for histological detection of the probe tracks by targeting ionized calcium-binding adapter molecule 1 (IBA-1), a protein expressed in microglia (Imai and Kohsaka, 2002) (Figures 3C,F). Both protocols reliably stained the probe tracks. Silicon probes were remotely advanced (50 μm in dCA1 and 100 μm in PL cortex) to monitor neuronal populations located at 4 different depths per animal (#1–4, Figure 3G). Our adjustment schedule ensured that the same dorsoventral (DV) coordinate was recorded in 3 consecutive days and that adjustments were performed 12–16 h before recording. Using the headgear system reported here, we acquired simultaneously PL and hippocampal spikes and LFP (Figure 3H). Next, we performed a representative unit analysis using the PL cortex data. Visual inspection of the raw traces revealed conspicuous spikes at all post-implantation days, both for new and reused silicon probes (Figure 4A). We decided to investigate the evolution of the signal to noise ratio (SNR) across days. Using a new probe and a reused probe (4th reuse), we observed no significant differences in the SNR in corresponding postoperative days (Figure 4B). However, there was a tendency for a progressive decline in the SNR across experimental days for each probe (Figure 4B). To further quantify and compare the performance of new and reused silicon probes, we estimated the total number of units per silicon probe across recording depths (Figures 4C,D). For that, we pooled the multi-units (MUs) and single-units (SUs) detected in 3 consecutive days per depth for 2 representative probes, specifically a new probe and a reused probes, in its 4th and 5th implantations (Figures 4C,D). Despite the variability of their yield, the new probe and the probe reused in 4 surgeries recorded comparable numbers of PL units. Upon implantation of the reused probe for the 5th time, we noted a sharp decline in the number of units acquired. However, while we excluded this probe from further attempts to collect unit data in the PL cortex, it should be noted that a 6th reusage would have been necessary to exclude that lower yield did not arise from other stochastic events (i.e., sub-optimal surgery, animal anatomy etc.), rather than a failed recovery. Together, these observations suggest that silicon probes can be effectively reused across animals to acquire MU and SU data. Lastly, since we recorded 3 consecutive days per depth, we decided to test whether the PL units were stable. We started by segregating the data from Figure 4D to compare the total number of units detected per day for each depth (Figure 4E). Per depth, we found different single units, as shown in Figure 4F by the differences in the autocorrelograms (ACGs) and waveforms. The daily yield tended to change even without adjustments, although strict stability in the number of units was sporadically observed (see Figure 4E; DVs #2 and #4 for the unused probe; #4 for the probe reused 4 times; and #2 and #3 for the probe reused 5 times). However, yield stability does not guarantee that the same PL cortex cells are being tracked, which prompted us to perform a stability analysis for PL SUs (see Methods). Despite the fluctuations in the number of units detected, we did find stable SUs spanning 2 and even 3 consecutive days in new and reused silicon probes (Figures 4G–I), suggesting that the drift observed was likely caused by intrinsic properties of the neuronal populations (i.e., representational drift (Hyman et al., 2012; Driscoll et al., 2017; Sauer et al., 2022)) and not by critical instability due to the surgery or headgear system.

### Representative spectral analysis in dCA1

**Figure 5:**
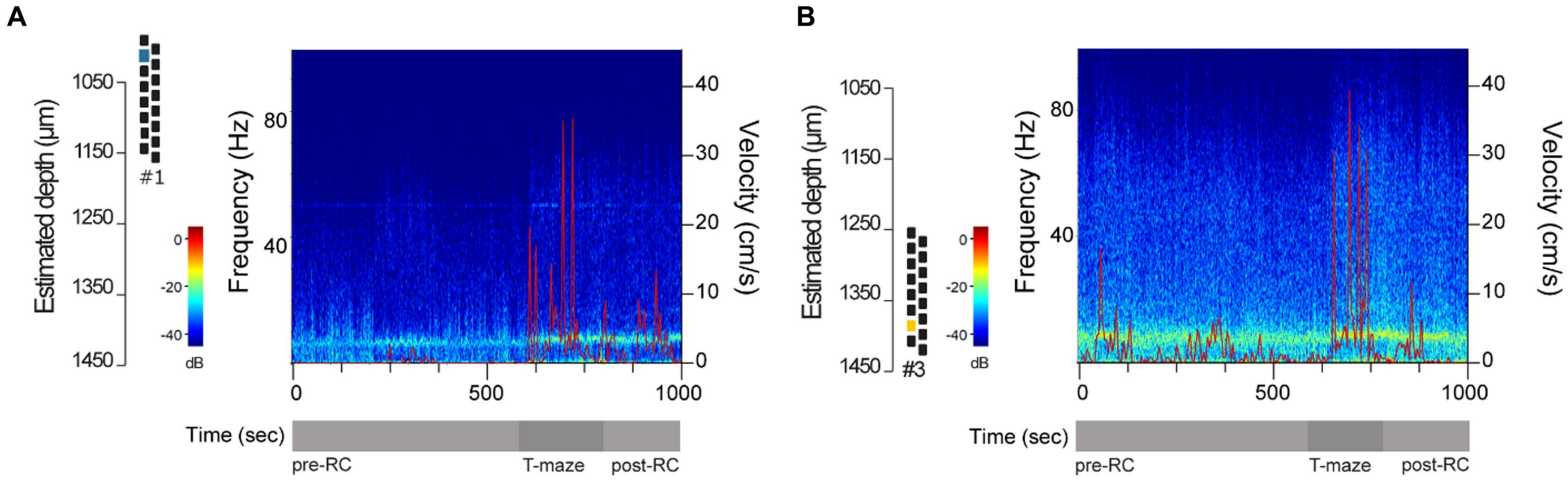
Representative spectral analysis in dCA1. **(A,B)** Representative spectrograms using a time interval that included periods of locomotion and immobility, based on the velocity (cm/s), in the recording cage before the behavioral test (pre-RC), in the T-maze during the first round of the spontaneous alternation test, and in the recording cage prior to starting the second alternation round (post-RC). LFP data were collected using a 16-channel silicon probe in its 5th reuse at post-op day 7 (DV#1) and 19 (DV#3). Power is color-coded and in the log scale (dB).

Since hippocampal rhythms are well established in rodent exploratory behavior (Vanderwolf, 1969; Colgin, 2016), we strengthened our performance assessment by performing a representative spectral analysis using dCA1 data from one reused silicon probe. For that, we computed discrete spectrograms from example epochs of locomotion and immobility based on the velocity at different recording depths. Visual inspection of the spectrograms revealed prominent theta oscillations (4–10 Hz) during periods of increased locomotor activity, mostly on the T-maze and in shorter epochs in the recording cage combined with short-lived, intermittent increases in gamma (30–80 Hz) oscillations (Figures 5A,B), matching the well described spectral properties of hippocampal activity in rodents performing spatial exploration in the recording cage and T-maze apparatus (Buzsáki, 2002; Colgin, 2016).

## Discussion

Silicon probes revolutionized *in vivo* electrophysiological recordings due to their increased sampling density (Buzsáki, 2004; Vandecasteele et al., 2012). However, their costs might be prohibitive and the standard protocols for silicon probe implantation limit their targeting flexibility. To mitigate these limitations, we implemented a 3-D printed headgear, which allowed multi-site recordings and silicon probe recovery and reuse in freely behaving mice. This is possible because our system combines a modular cap, capable of accommodating several nanodrives, with nanodrive’s encasements equipped with movable bases, for the reversible implantation of probes.

The implant strategy described in this work showed good performance in our quality control experiments. (1) The headgear was completely modular, decreasing the manual labor needed before and during surgeries. This simplification inherently reduced the burden on the animal, speeding up the recovery, as well as the burden on the experimenter. (2) The use of self-adhesive resin instead of rows of anchor screws and dental cement decreased the number of craniotomies performed, saving time during surgery and minimizing skull drilling. (3) The modular cap could accommodate, at least, 2 nanodrives. (4) The silicon probes implanted could be independently adjusted to maximize unit yield and targeting flexibility. (5) The headgear, which weighed 5.4 g on average, had no observable impact on behavior. (6) The implant was sturdy, showing no visible deterioration within a time window of weeks to months. (7) The headgear could record SU activity, MU activity, and LFP in 2 brain regions simultaneously, with significant stability. (8) Silicon probes could be effectively recovered and reused, showing SNR and unit yield comparable with new probes, within a certain number of re-implantation cycles (up to 7, in our hands).

Although the current design met our overall objectives, some problems remain unsolved and might be further optimized. This headgear could effectively support dual-site, independent recordings, but scaling up this system by adding extra nanodrives seems hard to achieve, particularly in mice. Two putative solutions would be the lateral expansion of the modular cap or the use of silicon probes with multiple shanks geometrically arranged to target multiple regions in the anterior–posterior and medial-lateral axes. Expanding the modular cap laterally would provide space for more nanodrives, ensuring our ability to independently adjust all the silicon probes implanted. This strategy is similar to tetrode-based implants, where each tetrode is driven by a screw secured to the cap (Yamamoto and Wilson, 2008; Liang et al., 2017). However, nanodrives are necessarily heavier, and, even if we could secure several nanodrives to the modular cap, certain combinations of brain targets might be impossible to achieve due to conflicts of space. Exploiting silicon probe geometry is an alternative solution. While this particular strategy was not pursued here, multi-shank probes do not allow independent adjustments and can only target neighboring brain regions. Besides these optimizations aiming at increasing the yield and targeting flexibility, additional improvements would focus on weight reduction, cap reusability, and merging this system with optogenetic and pharmacogenetic methodologies. Weight reduction is not straightforward, as all components were at their current miniaturization limit. Regarding headgear reusage, most parts were explanted and reimplanted between animals, except for the movable bases and the protective cap (circular base and lateral walls). Due to the relatively low costs of 3D printing, we decided to reinforce the protective cap with resin, to ensure the integrity and protection of the silicon probes. In future cohorts, we might upgrade the cap to ensure reversible closing in order to reuse the lateral walls, decreasing even further the costs with 3D printing. More importantly at this point will be the addition of optical fibers for optogenetics and/or cannulas for pharmacogenetics, allowing us to perform neural manipulation during *in vivo* recordings. However, new silicon probes with integrated μLEDs (Wu et al., 2015) offer a quick solution to bring manipulation capabilities to our system, without major changes in the configuration of the headgear. Ultimately, the implant described here is a proof of concept to perform independent, dual-site recordings in the PL cortex and dCA1. New combinations of brain targets and possible neuro-manipulation experiments will always require specific adaptations and case-by-case optimizations.

## Materials and methods

### Animals

Maintenance and handling of mice was performed according to the Animals Use and Care Guidelines issued by FELASA. All experiments were carried out according to the protocols approved by ORBEA 282 and 283 (Institutional Animal Welfare Body of the University of Coimbra), DGAV (Portuguese Regulatory Agency, reference number 8212/2021 and 8287/2021), and where in line with European Directives on Animal Welfare.

Mouse cages were kept in a temperature- and humidity-controlled room (22°C; 60% humidity), under a 12-h light/dark cycle (lights on at 7 AM/lights off at 7 PM). Mice were group-housed (4 in a cage) with *ad libitum* access to food and water. In this work, we used 10 male mice (4 non-implanted +6 implanted animals, 4–6 months old) of a PV-TdTomato-*Gprasp2* strain from our in-house breeding colony, with mean body weights of 35.26 ± 4.94 g. Enrichment was provided to all the cages and animals were handled 3 days before the experiments. Once implanted, mice were single housed for protection of the implant.

### Behavioral tasks

For the behavioral tasks, we used 4 non-implanted and 3 implanted mice (Supplementary Table S1, mice #4–6). Behavior and position data were acquired using CinePlex V3 Digital Video Recording and Tracking System (Plexon Inc., Dallas, TX) coupled with an Imaging Source™ Camera (640×480 resolution, 30fps). We took advantage of 2 behavioral tasks, specifically the delayed spontaneous alternation T-maze test, and the social T-maze test, which were performed 1 task/day for 3 consecutive days, for each recording depth in PL cortex and dCA1. On each experimental day, we plugged in the test mouse under anesthesia with isoflurane for 3 min (induction chamber: 3.5% isoflurane/O_2_ mixture, for induction; nosepiece: 1–2% isoflurane/O_2_ mixture, to plug in the animal) and performed a washout period in a recording cage for 30 min. After the washout period, the spontaneous locomotor activity of each animal was recorded for 10 min in the recording cage, and the animals performed the behavioral tasks, as described below. The distance traveled and velocity in these 10 min epochs were automatically quantified using Ethovision XT 11 (Noldus). For comparability purposes, non-implanted controls received the same treatment, including the 3 min anesthesia and the washout period. All tasks were performed on a T-maze (TS0701-M, OpenScience; 72×10 cm) with fresh bedding, positioned 52 cm above the floor, and under 25–30 lux, carefully cleaned with 70% ethanol and enzymatic spray (Men for San).

#### Delayed spontaneous alternation T-maze test

The spontaneous alternation test consisted of 5 rounds of spontaneous alternation, with 10 min epochs in the recording cage in-between rounds and a 10 min post-behavior epoch in the recording cage, before disconnecting the headgear. On each round, the test mouse was placed in the Start arm of the T-maze and allowed to explore the maze. Once the animal chose one of the Goal arms (considered when its body fully entered the arm), a movable door was used to enclose the animal in that Goal arm for 30 s, after which the test mouse was gently returned and enclosed in the Start arm. After 30 s in the Start arm, the movable door was lifted and the animal was allowed to freely choose one of the Goal arms. Performance was quantified as the percentage of spontaneous alternation between Goal arms.

#### Social T-maze test

In this test, we placed plexiglass barriers at the end of the Goal arms to create 2 social chambers for the presentation of stimulus animals. Interactions between the test mouse and the stimulus animals were possible through holes in the barriers. The test subject was acclimatized to the T maze for 10 min. In this epoch, the social chambers were empty. After acclimatization, the animal was briefly enclosed in the Start arm, the first stimulus animal was randomly placed in one of the social chambers, and the test subject was allowed to re-explore the maze for 10 min. After another brief enclosure, the second stimulus animal was placed in the remaining social chamber, and the test subject re-explored the maze for 10 min. The session ended with a final 10 min epoch similar to the acclimatization period and a subsequent 10 min post-behavior epoch in the recording cage, before disconnecting the headgear. For each test mouse, we computed the time spent in each compartment using CinePlex Editor (Plexon).

### Preparation of the headgear

Our headgear was based on Vöröslakos and colleagues (Vöröslakos et al., 2021), with several modifications (Figure 1 and Results), and consisted of a protective cap and two nanodrives in their encasements (see Supplementary Table S2 for key resources and Supplementary Table S3 for troubleshooting).

#### Fabrication

The parts of the headgear were 3D printed using a Stratasys Objet260 3D printer and the PolyJet technology. PolyJet involves the precise deposition of tiny droplets of liquid photopolymer materials onto a build platform, followed by their instant curing via UV light. This manufacturing technique shows a level of precision and detail that is challenging to achieve with alternative manufacturing methods, creating small and intricate components with resolutions below 0.4 mm. Regarding the 3D printing material, we used VeroWhitePlus RGD835 resin, due to its lightweight and durability, making it an ideal candidate for preserving the intricate details of the design, while meeting the stringent demands of the application.

#### Protective cap

The protective cap was composed of a circular base and 2 lateral walls, following the CAD files available in Vöröslakos et al. (2021), with in-house adaptations (Figure 1 and Results – modification #1). To prepare the protective cap, the lateral walls were covered with light aluminum mesh by gluing it with self-adhesive resin (Maxcem Elite). Two male header pins were inserted in the two through-holes available in the anterior and posterior limits of the lateral walls and glued with cyanoacrylate, to serve as soldering points for a third male header pin. The third male header pin was soldered horizontally to support the Omnetics connectors. Once assembled, the lateral walls formed an incomplete cage-like structure, protecting the silicon probes and providing horizontal posts to attach the Omnetics connectors.

#### Nanodrives and encasements

The encasements for the metal nanodrives were composed of a printed frame and a detachable base, based on the CAD files available in Vöröslakos et al. (2021), with in-house adaptations (Figure 1 and Results – modification#2 and #3). To prepare the encasements, we attached the encasements’ frames and the detachable bases using stainless steel screws. The nanodrives were fitted inside the frames, carefully glued with cyanoacrylate - without touching the nanodrive shuttle - and secured to stereotaxic holders.

#### Silicon probes

Before unboxing the silicon probes, we glued male header pins onto the anterior walls of the Omnetics connectors using self-adhesive resin, to support the connectors onto the horizontal male header pins on top of the protective cap. Next, we soldered together the ground and reference wires of each silicon probe onto a common ground wire (Phoenix Wire, Inc). Silicon probes were unboxed following the manufacturer’s instructions, and carefully attached to the shuttles of the nanodrives using cyanoacrylate, with minimal orientation biases. The Omnetics connectors were fixed to the stereotaxic holders using tape.

### Surgery

We implanted 6 mice (Supplementary Table S1, mice #1–6) with 2 independently movable silicon probes (Cambridge Neurotech, P-series, 16 channels) to record spikes and LFP simultaneously from the deep layers of the PL cortex (coordinates: AP, 1.6 mm from bregma; ML, 0.4 mm from the midline; DV, 1.5 mm from the surface of the brain) and from dCA1 (coordinates: AP, 2.1 mm from bregma; ML, 1.0 mm from the midline; DV, 1.0 mm from the surface of the brain).

#### Anesthesia and pre-incision procedures

Right before the surgery, we disinfected the surgical area with 95% ethanol, and sterilized the surgical material by autoclaving. Mice were weighted and placed inside an induction chamber under a 3.5% isoflurane/O2 mixture. When fully anesthetized, mice were transferred to the stereotaxic apparatus, where they were ventilated through a nosepiece. Fixation was performed using non-rupture ear bars to protect the tympanic membranes. After successful fixation, we lowered the level of anesthesia (2% isoflurane), administered meloxicam (1 mg/kg, intraperitoneal) and buprenorphine (0.05 mg/kg, subcutaneous), applied ophthalmic ointment (Clorocil®, Laboratório Edol), and shaved the mice to expose the skull’s skin. The hairless skin was cleaned with a cotton swab immersed in Povidone-Iodine 10% topical solution, followed by 70% ethanol. This cleaning procedure was repeated 3 times, using a circular movement from the center to the periphery.

#### Incision, skull cleaning and marking

Using a scalpel and a dissecting microscope at a low magnification (10x to 20x), we made a median incision in the scalp from the level of the eyes to the back of the skull. The skin and soft tissues were separated from the skull by gentle scraping with cotton swabs, and 4 bulldog clips were attached to the subcutaneous tissue, to pull the skin sidewise and create a rectangular surgical window. Using the scalpel, we scraped the periosteum from the surface of the skull, cleaned it with saline solution, stopped any bleeding, and let the skull dry. Once bregma and lambda were clearly seen, their z-coordinates were matched to level the animals’ heads in the horizontal plane. This leveling step was repeated in the mediolateral axis. The coordinates of bregma and lambda, the craniotomies, the position of the 2 anchor screws (coordinates: AP, 5.7 mm from bregma; ML, −2.1 mm from the midline; and AP, 5.7 mm from bregma; ML, 1.3 mm from the midline), and the position of the common ground wires (coordinates: AP, 5.9 mm from bregma; ML, −0.8 mm from the midline) were measured and marked with a glass micropipette filled with gentian violet.

#### Craniotomies and screw placement

Using a hand-held high-speed drill (drill bit size of 0.2 mm, C1.104.010, Edenta), we performed the craniotomies. For that, the high-speed drill was held perpendicular to the skull under gentle pressure. Bone removal was checked using a 26 G needle. Next, we drilled the holes for the anchor screws and for the common ground wires in the skull above the cerebellum, after which we placed the anchor screws. When driving the screws, we performed the minimum amount of turns to get them stable but allowed a margin of about 0.5 mm. Screw stability was tested by gently shaking it with a pair of tweezers, and was accepted when the skull moved as a whole with the screw.

#### Attachment of the circular base and durotomies

Having finished all the drilling steps, we attached the circular base to the skull, minimizing the exposure of the resin to high-frequency vibrations. The base was held in place, while self-adhesive resin (Relyx™ Unicem 2 Automix) was progressively applied along the inner contact line between the circular base, the outer ridge of the skull, and the anchor screws, to create a sealed area. The resin was applied under dim light, to slow down the curation process, and cured using UV light. Next, the *dura mater* was removed using a hook-shaped 26 G needle, bent against a hard surface. Efforts were made not to damage blood vessels or the *superior sagittal sinus*. In case of bleeding, we applied OctoColagen (Laboratorios Clarben SA) under gentle pressure and cleaned with saline solution, once the bleeding stopped. After durotomy, the cranial windows were kept hydrated with saline solution, and the coordinates of the silicon probes within each window were confirmed.

#### Probe implantation

To facilitate visual control during probe implantation, we started in the PL cortex, after which we implanted the second probe in dorsal CA1. We attached the first stereotaxic holder to the stereotaxic apparatus and painted the silicon probe using DiI, by gently dipping the probe in a microtube filled with the staining solution and letting it dry for about 20 s. The painting procedure was repeated twice. The probe was positioned above the PFC (see Figure 1 for encasement’s position and orientation) and was lowered at a rate of about 0.5 mm/min, under visual control using the dissecting microscope. The craniotomy was kept hydrated with saline solution throughout the entire implant procedure. Once reached the desired depth, the movable base of the microdrive was secured to the skull and to the circular base using self-adhesive resin. The first stereotaxic holder was then removed to bring the second one. For that, the Omnetics connector attached to the first holder with tape was carefully transferred to a temporary supporting point, typically one of the tubing connected to the breather’s nosepiece, and the tape was replaced. To implant the second silicon probe, we replicated the sequence of steps described above. Having finished the second implant, we transferred the Omnetics connector of the first probe from the temporary supporting point to the holder, such that the 2 connectors of the 2 probes became attached to the same holder with tape. While lowering the silicon probes, sometimes the movable base would touch the skull or the circular base before the silicon probe had reached the desired DV coordinate, because the movable base had a height of 1 mm, adding extra distance between the probe and the final DV coordinate. When this happened, we lifted the probe, detached the holder from the stereotaxic apparatus, removed the tape attached to the Omnetics connector, and made compensatory turns in the nanodrive to lower the silicon probe by ~1–1.5 mm. Then, the implantation was resumed. Alternatively, if the probe was close to the target (typically <0.3 mm), we proceeded with the implantation and lowered the silicon probe later, during the recovery period, using the nanodrive. This avoided additional intra-operatory manipulation of the probes, which can be damaging and add extra time to the surgery. In our hands, the compensatory turns could also be tentatively performed on the previous day, while preparing the surgery. However, due to variability when gluing the silicon probes to the shuttles of the nanodrives, intra-operatory adjustments might still be needed to optimize the dynamic range.

#### Placing of the common ground wires and closure of the headgear

The common ground wires of the 2 probes were independently inserted in the craniotomy above the cerebellum, and the craniotomy was sealed with self-adhesive resin. To finally close the headgear, we attached the lateral walls to the rails in the circular base and secured them together with 3 wires. Small drops of self-adhesive resin were added in the interfaces between the lateral walls, and between the lateral walls and the circular base. The Omnetics connectors were sequentially detached from the holder, and secured to the horizontal male header pins on top of the lateral walls using drops of self-adhesive resin. To finish the surgery, we covered the headgear using tape and Parafilm M(R), turned off the anesthesia, and released the animal from the ear bars.

#### Postoperative care

At the end of the surgery, mice were weighed to determine the weight of the headgear, were hydrated with Ringer’s lactate solution (B. Braun Vet Care), and were allowed to recover on the heating pad, after which they were transferred to a new home cage with soft food (hydrated pellets and peanut butter), regular pellets, and water with minocycline (2 mg/mL, 13,614–98-7 Acros). To monitor the recovery, we recorded the animal’s weight, water consumption, food consumption, motor activity, and signs of pain on a daily basis.

### Electrophysiological recordings

The post-surgical recovery was 7–14 days. Since mice showed variability in their recovery, we implemented criteria to start the recordings, which consisted of at least 7 days post-surgery and 3 consecutive days without weight loss. We checked the quality of the traces by plugging in the animals for short periods before completing the recovery, but never before the 5th day post-surgery, and moved the probes (50 to 100 μm/ day) until they reached the deep layers of the PL cortex and the pyramidal layer of dCA1. The recordings started when we reached the target regions and detected significant unit activity. The pyramidal layer of CA1 was identified upon detection of sharp-wave ripples.

Electrophysiological data were acquired, amplified, and digitized at 40 kHz using the Omniplex Neural Recording Data Acquisition System (Plexon, Inc., Dallas, TX), with 2 independent headstages (HST/16o25-GEN2-18P-2GP-G1-2LED, Plexon, Inc., Dallas, TX), and 2 ultra-fine cables (HSC/16o25-GEN2-ufw-36 L, Plexon, Inc., Dallas, TX). Behavioral data were acquired using the CinePlex V3 Digital Video Recording and Tracking System (Plexon Inc., Dallas, TX) coupled with an Imaging Source TM camera (640×480 resolution, 30 fps). We acquired both the movies for *post-hoc* analysis and the xy position using the LEDs mounted onto the headstages. On each recording day, we stored 1 PLX file with raw neuronal data and 1 AVI file with behavioral data aligned in time with the neuronal data. Mice did not carry the headstages in their home cage and were briefly anesthetized with isoflurane using the induction chamber and the nosepiece (see Surgery) before and after the recording sessions. We anesthetized them before the sessions to plug in the headstages and ultra-fine cables, and after the sessions to unplug the headstages and adjust the silicon probes. Adjustments were made (typically 50 μm in dCA1 and 100 μm in PL cortex) to record from the same DV coordinate in 3 consecutive days. All animals underwent a washout period of 30 min before starting the recording sessions to mitigate the impact of isoflurane on the recordings.

### Silicon probe recovery and reuse

To recover the silicon probes, we anesthetized the mice with isoflurane and placed them in the stereotaxic apparatus, as described for the surgery. We started by cutting the common ground wires, secured one of the nanodrives to the stereotaxic holder, and detached the corresponding Omnetics connector by cutting the resin connecting the Omnetics to the horizontal male header pin at the top of the headgear. The connector was attached to the stereotaxic holder with tape and the nanodrive was recovered by unscrewing the encasement’s frame from the movable base. The procedure was repeated for the second nanodrive.

To reuse the silicon probes, we immersed them in 1% Tergazyme overnight, at room temperature to clean biological debris, followed by an immersion in H_2_O (~12 h) to remove the cleaning solution. Reused silicon probes had different pre-surgery procedures, requiring only the addition of a new movable base to the encasement’s frame and the extension of the common ground wire.

### Histological confirmation of the recording sites

After recovering the probes, mice were deeply anesthetized and sequentially perfused with 1X phosphate buffered saline (PBS) and 4% paraformaldehyde (PFA). Brains were extracted, fixated overnight in 4% PFA, and stored in 30% sucrose. Using a vibratome (Leica VT1200s, Leica Microsystems, USA), we cut 50 μm coronal slices containing dCA1 and PL cortex. Selected slices were sequentially washed 5 times in 1X PBS for 10 min, incubated in blocking buffer (10% normal goat serum (NGS) and 0.4% triton in 1X PBS) for 1 h at room temperature, washed in 1X PBS, and incubated with primary antibody (rabbit anti-IBA-1 polyclonal primary antibody, 1:500, 019–19,741, Wako) in antibody blocking solution (5% NGS in 1X PBS) for 16 h at 4°C. Slices were then washed 3 times in 1X PBS for 20 min, incubated with secondary antibody (goat anti-rabbit Alexa 488, 1:1000, A11008 - Life Technologies) in antibody blocking solution for 2 h at room temperature, and the washing step was repeated. Sections were mounted in Vectashield (VECTASHIELD HardSet Mounting Medium with DAPI (Vector) H-1500) and imaged in a 710 LSM confocal microscope (Carl Zeiss), with 20× (0.8 NA) and 40× (1.4 NA) objectives. Fluorophores were excited using a 405 nm diode, a 488 nm argon, a 561 nm diode-pumped solid-state (DPSS). Detection intervals were set at (nm) 409–468 (DAPI), 497–574 (Alexa 488), 584–642 (tdTomato). We identified the probe tracks as DiI and IBA-1 positive linear lesions in dCA1 and PL cortex.

### Electrophysiological data processing

#### Spike sorting

Spike sorting was performed semi-automatically with Offline Sorter V3.3.5 (Plexon). For spike detection and waveform extraction, wideband data were band-pass filtered between 250 Hz and 8 kHz using a 4th-order Butterworth filter, and thresholded at −3 standard deviations. Detected spikes were discriminated with principal component analysis and semi-automatically sorted using the T-distribution Expectation–Maximization (E-M) clustering algorithm (scan over a range of 1–3 degrees of freedom, with a step of 5 in the 3D feature space), followed by manual curation. ACGs were computed over 0.2 s of spike data with 5 ms bins (NeuroExplorer V4, Plexon). Units were classified as a good single units (SUs) if they matched the following criteria: (1) physiological waveform, significantly above the noise level on visual inspection; (2) minimum firing rate of 0.5 Hz (i.e., at least 300 spikes in 600 s); and (3) <1% of inter-spike intervals (ISIs) below 2 ms in the ACGs. Units were classified as multi-units (MUs) if they only matched criteria (1) and (2). For good SUs, we removed spikes with inter-spike intervals below 1 ms (Offline Sorter V3.3.5; ‘Remove Short ISI Waveforms’).

#### Unit estimation per channel

We estimated the total number of units per channel per recording depth by combining all SUs and MUs detected in that channel over 3 consecutive days for each depth.

#### Single units stability

To assess SU stability across 3 consecutive days at the same depth, one of the days was used as the template file. This file was sorted and manually curated, and the sorting criteria were saved in a TPL file. The TPL file was then used to sort each of the remaining days. SUs present in all the files, with matching waveforms and ACGs were considered stable. ACGs were computed as described above. The waveform data were extracted from NeuroExplorer and displayed in GraphPad V8.

#### Spectrograms

For LFP analysis, wideband data were band-pass filtered between 0.3 Hz and 200 Hz. Spectrograms were computed in NeuroExplorer V4 (Plexon) using discrete time intervals that included both periods of walking/running and periods of quiet wakefulness in the recording cage before the behavioral test, in the T-maze during the behavioral test, and in the recording cage after the behavioral test. Spectrograms were displayed as the log of PSD (dB), with 1,024 frequency values and a maximum frequency of 200 Hz, and were smoothed with a Gaussian filter (width 5), animal tracking and velocity were performed using Bonsai (Lopes et al., 2015).

#### Signal to noise ratio (SNR)

The SNR was calculated for each channel containing units using the Calculate SNR tool from Offline Sorter V4. This function computes the SNR as (sigma squared within signal)/(sigma squared within noise). In our estimation, the signal corresponded to the sorted spikes (excluding unsorted or invalid spikes), while the noise was sampled from interspike segments.

## Data availability statement

The original contributions presented in the study are included in the article/Supplementary material, further inquiries can be directed to the corresponding authors.

## Ethics statement

The animal study was approved by ORBEA Institutional Animal Welfare Body of the University of Coimbra/CNC. The study was conducted in accordance with the local legislation and institutional requirements.

## Author contributions

EF-F: Conceptualization, Data curation, Formal analysis, Funding acquisition, Investigation, Methodology, Software, Supervision, Writing – original draft, Writing – review & editing. ML: Data curation, Formal analysis, Investigation, Methodology, Writing – original draft, Writing – review & editing. TR: Data curation, Formal analysis, Investigation, Methodology, Writing – original draft, Writing – review & editing. BC: Investigation, Writing – review & editing. PF: Investigation, Writing – review & editing. PM: Visualization, Writing – review & editing. JV: Resources, Writing – review & editing. MT: Resources, Writing – review & editing. CK: Writing – review & editing. JP: Conceptualization, Funding acquisition, Project administration, Resources, Supervision, Writing – original draft, Writing – review & editing.

## References

[ref1] Adrian E. D. Moruzzi G. (1939). Impulses in the pyramidal tract. J. Physiol. 97, 153–199. doi: 10.1113/jphysiol.1939.sp003798, PMID: 16995153 PMC1393899

[ref2] Blanche T. J. Spacek M. A. Hetke J. F. Swindale N. V. (2005). Polytrodes: high-density silicon electrode arrays for large-scale multiunit recording. J. Neurophysiol. 93, 2987–3000. doi: 10.1152/jn.01023.2004, PMID: 15548620

[ref3] Brosch M. Vlasenko A. Ohl F. W. Lippert M. T. (2021). TetrODrive: an open-source microdrive for combined electrophysiology and optophysiology. J. Neural Eng. 18:046030. doi: 10.1088/1741-2552/abf608, PMID: 33908896

[ref4] Buzsáki G. (2002). Theta oscillations in the hippocampus. Neuron 33, 325–340. doi: 10.1016/S0896-6273(02)00586-X, PMID: 11832222

[ref5] Buzsáki G. (2004). Large-scale recording of neuronal ensembles. Nat. Neurosci. 7, 446–451. doi: 10.1038/nn1233, PMID: 15114356

[ref6] Buzsáki G. Anastassiou C. A. Koch C. (2012). The origin of extracellular fields and currents--EEG, ECoG, LFP and spikes. Nat. Rev. Neurosci. 13, 407–420. doi: 10.1038/nrn3241, PMID: 22595786 PMC4907333

[ref7] Chung J. Sharif F. Jung D. Kim S. Royer S. (2017). Micro-drive and headgear for chronic implant and recovery of optoelectronic probes. Sci. Rep. 7:2773. doi: 10.1038/s41598-017-03340-5 28584246 PMC5459843

[ref8] Colgin L. L. (2016). Rhythms of the hippocampal network. Nat. Rev. Neurosci. 17, 239–249. doi: 10.1038/nrn.2016.21, PMID: 26961163 PMC4890574

[ref9] Csicsvari J. Henze D. A. Jamieson B. Harris K. D. Sirota A. Barthó P.. (2003). Massively parallel recording of unit and local field potentials with silicon-based electrodes. J. Neurophysiol. 90, 1314–1323. doi: 10.1152/jn.00116.2003, PMID: 12904510

[ref10] Driscoll L. N. Pettit N. L. Minderer M. Chettih S. N. Harvey C. D. (2017). Dynamic reorganization of neuronal activity patterns in parietal cortex. Cells 170, 986–999.e16. doi: 10.1016/j.cell.2017.07.021, PMID: 28823559 PMC5718200

[ref11] Edfawy M. Guedes J. R. Pereira M. I. Laranjo M. Carvalho M. J. Gao X.. (2019). Abnormal mGluR-mediated synaptic plasticity and autism-like behaviours in Gprasp2 mutant mice. Nat. Commun. 10:1431. doi: 10.1038/s41467-019-09382-9, PMID: 30926797 PMC6440958

[ref12] Ferreira-Fernandes E. Pinto-Correia B. Quintino C. Remondes M. (2019). A gradient of hippocampal inputs to the medial Mesocortex. Cell Rep. 29, 3266–3279.e3. doi: 10.1016/j.celrep.2019.11.011, PMID: 31801088

[ref13] Gray C. M. Maldonado P. E. Wilson M. McNaughton B. (1995). Tetrodes markedly improve the reliability and yield of multiple single-unit isolation from multi-unit recordings in cat striate cortex. J. Neurosci. Methods 63, 43–54. doi: 10.1016/0165-0270(95)00085-2, PMID: 8788047

[ref14] Grinvald A. Hildesheim R. (2004). VSDI: a new era in functional imaging of cortical dynamics. Nat. Rev. Neurosci. 5, 874–885. doi: 10.1038/nrn1536, PMID: 15496865

[ref15] Guardamagna M. Eichler R. Pedrosa R. Aarts A. Meyer A. F. Battaglia F. P. (2022). The hybrid drive: a chronic implant device combining tetrode arrays with silicon probes for layer-resolved ensemble electrophysiology in freely moving mice. J. Neural Eng. 19:036030. doi: 10.1088/1741-2552/ac6771, PMID: 35421850

[ref16] Headley D. B. DeLucca M. V. Haufler D. Paré D. (2015). Incorporating 3D-printing technology in the design of head-caps and electrode drives for recording neurons in multiple brain regions. J. Neurophysiol. 113, 2721–2732. doi: 10.1152/jn.00955.2014, PMID: 25652930 PMC4416572

[ref17] Hyman J. M. Ma L. Balaguer-Ballester E. Durstewitz D. Seamans J. K. (2012). Contextual encoding by ensembles of medial prefrontal cortex neurons. Proc. Natl. Acad. Sci. U. S. A. 109, 5086–5091. doi: 10.1073/pnas.1114415109, PMID: 22421138 PMC3323965

[ref18] Imai Y. Kohsaka S. (2002). Intracellular signaling in M-CSF-induced microglia activation: role of Iba1. Glia 40, 164–174. doi: 10.1002/glia.10149, PMID: 12379904

[ref19] Jun J. J. Steinmetz N. A. Siegle J. H. Denman D. J. Bauza M. Barbarits B.. (2017). Fully integrated silicon probes for high-density recording of neural activity. Nature 551, 232–236. doi: 10.1038/nature24636, PMID: 29120427 PMC5955206

[ref20] Kim H. Brünner H. S. Carlén M. (2020). The DMCdrive: practical 3D-printable micro-drive system for reliable chronic multi-tetrode recording and optogenetic application in freely behaving rodents. Sci. Rep. 10:11838. doi: 10.1038/s41598-020-68783-9 32678238 PMC7366717

[ref21] Kim K. Vöröslakos M. Seymour J. P. Wise K. D. Buzsáki G. Yoon E. (2020). Artifact-free and high-temporal-resolution in vivo opto-electrophysiology with microLED optoelectrodes. Nat. Commun. 11:2063. doi: 10.1038/s41467-020-15769-w, PMID: 32345971 PMC7188816

[ref22] Kipke D. R. Shain W. Buzsáki G. Fetz E. Henderson J. M. Hetke J. F.. (2008). Advanced neurotechnologies for chronic neural interfaces: new horizons and clinical opportunities. J. Neurosci. 28, 11830–11838. doi: 10.1523/JNEUROSCI.3879-08.2008, PMID: 19005048 PMC3844837

[ref23] Kozai T. D. Y. Vazquez A. L. Weaver C. L. Kim S.-G. Cui X. T. (2012). In vivo two-photon microscopy reveals immediate microglial reaction to implantation of microelectrode through extension of processes. J. Neural Eng. 9:066001. doi: 10.1088/1741-2560/9/6/066001, PMID: 23075490 PMC3511663

[ref24] Liang L. Oline S. N. Kirk J. C. Schmitt L. I. Komorowski R. W. Remondes M.. (2017). Scalable, lightweight, integrated and quick-to-assemble (SLIQ) Hyperdrives for functional circuit dissection. Front. Neural Circuits 11:8. doi: 10.3389/fncir.2017.00008, PMID: 28243194 PMC5303737

[ref25] Lopes G. Bonacchi N. Frazão J. Neto J. P. Atallah B. V. Soares S.. (2015). Bonsai: an event-based framework for processing and controlling data streams. Front. Neuroinform. 9:7. doi: 10.3389/fninf.2015.00007, PMID: 25904861 PMC4389726

[ref26] Lu L. Popeney B. Dickman J. D. Angelaki D. E. (2018). Construction of an improved multi-tetrode Hyperdrive for large-scale neural recording in behaving rats. J. Vis. Exp. 135:57388. doi: 10.3791/57388 PMC6101149 29806835

[ref27] McNaughton B. L. O’Keefe J. Barnes C. A. (1983). The stereotrode: a new technique for simultaneous isolation of several single units in the central nervous system from multiple unit records. J. Neurosci. Methods 8, 391–397. doi: 10.1016/0165-0270(83)90097-3, PMID: 6621101

[ref28] Newton T. H. Reimann M. W. Abdellah M. Chevtchenko G. Muller E. B. Markram H. (2021). In silico voltage-sensitive dye imaging reveals the emergent dynamics of cortical populations. Nat. Commun. 12:3630. doi: 10.1038/s41467-021-23901-7, PMID: 34131136 PMC8206372

[ref29] Nitzan N. McKenzie S. Beed P. English D. F. Oldani S. Tukker J. J.. (2020). Propagation of hippocampal ripples to the neocortex by way of a subiculum-retrosplenial pathway. Nat. Commun. 11:1947. doi: 10.1038/s41467-020-15787-8, PMID: 32327634 PMC7181800

[ref30] O’Keefe J. Dostrovsky J. (1971). The hippocampus as a spatial map. Preliminary evidence from unit activity in the freely-moving rat. Brain Res. 34, 171–175. doi: 10.1016/0006-8993(71)90358-1, PMID: 5124915

[ref31] Rein B. Ma K. Yan Z. (2020). A standardized social preference protocol for measuring social deficits in mouse models of autism. Nat. Protoc. 15, 3464–3477. doi: 10.1038/s41596-020-0382-9, PMID: 32895524 PMC8103520

[ref32] Sauer J.-F. Folschweiller S. Bartos M. (2022). Topographically organized representation of space and context in the medial prefrontal cortex. Proc. Natl. Acad. Sci. U. S. A. 119:e2117300119. doi: 10.1073/pnas.2117300119, PMID: 35121665 PMC8833199

[ref33] Sun C. Cao Y. Huang J. Huang K. Lu Y. Zhong C. (2022). Low-cost and easy-fabrication lightweight drivable electrode array for multiple-regions electrophysiological recording in free-moving mice. J. Neural Eng. 19:016003. doi: 10.1088/1741-2552/ac494e, PMID: 34996053

[ref34] Vandecasteele M. M S. Royer S. Belluscio M. Berényi A. Diba K.. (2012). Large-scale recording of neurons by movable silicon probes in behaving rodents. J. Vis. Exp. 61:e3568. doi: 10.3791/3568, PMID: 22415550 PMC3399468

[ref35] Vanderwolf C. H. (1969). Hippocampal electrical activity and voluntary movement in the rat. Electroencephalogr. Clin. Neurophysiol. 26, 407–418. doi: 10.1016/0013-4694(69)90092-3, PMID: 4183562

[ref36] Voigts J. Newman J. P. Wilson M. A. Harnett M. T. (2020). An easy-to-assemble, robust, and lightweight drive implant for chronic tetrode recordings in freely moving animals. J. Neural Eng. 17:026044. doi: 10.1088/1741-2552/ab77f9, PMID: 32074511 PMC8878001

[ref37] Voigts J. Siegle J. Pritchett D. Moore C. (2013). The flexDrive: an ultra-light implant for optical control and highly parallel chronic recording of neuronal ensembles in freely moving mice. Front. Syst. Neurosci. 7:8. doi: 10.3389/fnsys.2013.00008, PMID: 23717267 PMC3652307

[ref38] Vöröslakos M. Petersen P. C. Vöröslakos B. Buzsáki G. (2021). Metal microdrive and head cap system for silicon probe recovery in freely moving rodent. elife 10:e65859. doi: 10.7554/eLife.65859, PMID: 34009122 PMC8177890

[ref39] Wilson M. A. McNaughton B. L. (1993). Dynamics of the hippocampal ensemble code for space. Science 261, 1055–1058. doi: 10.1126/science.8351520, PMID: 8351520

[ref40] Wise K. D. Najafi K. (1991). Microfabrication techniques for integrated sensors and microsystems. Science 254, 1335–1342. doi: 10.1126/science.1962192, PMID: 1962192

[ref41] Wu F. Stark E. Ku P. C. Wise K. D. Buzsáki G. Yoon E. (2015). Monolithically integrated μLEDs on silicon neural probes for high-resolution optogenetic studies in behaving animals. Neuron 88, 1136–1148. doi: 10.1016/j.neuron.2015.10.032, PMID: 26627311 PMC4702503

[ref42] Yamamoto J. Wilson M. A. (2008). Large-scale chronically implantable precision motorized microdrive array for freely behaving animals. J. Neurophysiol. 100, 2430–2440. doi: 10.1152/jn.90687.2008, PMID: 18667539 PMC2576215

