# Supplementary material for: In vivo recordings in freely behaving mice using independent silicon probes targeting multiple brain regions
Source: Front Neural Circuits. 2023 Dec 22;17:1293620. doi: 10.3389/fncir.2023.1293620 (PMC10771849; doi:10.3389/fncir.2023.1293620)
Supplement: Supplementary file 1 [file Table_1.docx]

***Supplementary Material - Tables***

***In vivo* recordings in freely behaving mice using independent silicon probes targeting multiple brain regions**

Emanuel Ferreira-Fernandes, Mariana Laranjo, Tiago Reis, Bárbara Canijo, Pedro A. Ferreira, Pedro Martins, João Vilarinho, Mahmoud Tavakoli, Carolina Kunicki, João Peça

**Table 1.** Probe recovery and reuse across animals. Overview of silicon probe implantation experiments. Mice were dual implanted in the PL cortex and dCA1. Mice #7 and #8 are unpublished animals from additional implantation experiments and serve only to further reinforce the effectiveness of our protocols.

| **Animal** | **Brain Region** | **Implanted period** | **Probe** | **Outcome** |
| --- | --- | --- | --- | --- |
| #1-3 | *PL* | maximum 1 week | Probe 1 | Intact |
|  | *dCA1* |  | Probe 2 | Intact |
| #4 | *PL* | *4 weeks* | Probe 1 | Intact |
|  | *dCA1* |  | Probe 2 | Intact |
| #5 | *PL* | *4 weeks* | Probe 1 | Intact |
|  | *dCA1* |  | Probe 2 | Red cable broke (lost) |
| #6 | *PL* | *4 weeks* | *Probe 3* | Intact |
|  | *dCA1* |  | Probe 1 | Intact |
| #7 | *PL* | *4 weeks* | *Probe 3* | Broken (lost) |
|  | *dCA1* |  | Probe 1 | White cable broke (lost) |
| #8 | *PL* | *4 weeks* | *Probe 4* | Broken (lost) |
|  | *dCA1* |  | *Probe 5* | Intact |

|  | | **Probe 1** | **Probe 2** | **Probe 3** | **Probe 4** | **Probe 5** |
| --- | --- | --- | --- | --- | --- | --- |
| **Number of uses** | ***PL*** | 3+2 | 3+2 | 2 | 1 | - |
|  | ***dCA1*** | *2* | *-* | - | - | 1 |
| **Total** | | *7* | *5* | 2 | 1 | 1 |

**Table 2.** Key resources table.

| **Product** | **Source/Brand** | **Comments/Link** |
| --- | --- | --- |
| ***Behavior and electrophysiology recordings*** | | |
| CinePlex V3 Digital Video Recording and Tracking System | Plexon Inc., Dallas | <https://plexon.com/wp-content/uploads/2020/01/Cineplex-Studio-User-Guide.pdf> |
| CinePlex Editor | Plexon Inc., Dallas | <https://plexon.com/wp-content/uploads/2017/06/CinePlex-Editor-User-Guide.pdf> |
| Imaging Source™ Camera |  | 640x480 resolution, 30 frames per sec |
| Ethovision XT 11 | Noldus | <https://www.noldus.com/ethovision-xt> |
| T-maze TS0701-M | OpenScience | <http://www.openscience.ru/index.php?page=ts&item=006&lang=en> |
| Enzymatic spray | Menforsan | <https://menforsan.com/en/enviroments-products/258-enzimatic-urine-and-stain-remover-750ml.html> |
| Implantable nano-Drive  2x4x10 mm; ~7 mm travel; fiber optic compatible  Nano-Drive V2 | Cambridge Neurotech | <https://www.cambridgeneurotech.com/nanodrives> |
| Chronic 16 channel P-1 probe with Omnetics connector  1 shank, 6 mm  length ASSY-79 P-1 | Cambridge Neurotech | <https://www.cambridgeneurotech.com/assets/files/ASSY-79-P-1-P-2-map.pdf> |
| Stereotaxic adaptor for holding Nano-Drive | Cambridge Neurotech | <https://www.cambridgeneurotech.com/catalog> |
| Headstages HST/16o25-GEN2-18P-2GP-G1-2LED | Plexon, Inc., Dallas, TX | <https://plexon.com/wp-content/uploads/2020/01/Headstages-Datasheet_0.pdf> |
| Ultra-fine cables HSC/16o25-GEN2-ufw-36L | Plexon, Inc., Dallas, TX | <https://plexon.com/wp-content/uploads/2020/01/Headstages-Datasheet_0.pdf> |
| ***Protective cap*** | | |
| Dental Cement Maxcem Elite | Kerr | <https://www.kerrdental.com/kerr-restoratives/maxcem-elite-self-etch-self-adhesive-resin-dental-cement> |
| CAD files |  | https://github.com/ncblcnc/Social-Code |
| Cyanoacrylate | Super Glue gel | **-** |
| Ground wire | Phoenix Wire, Inc | <https://www.phoenixwireinc.com/ptfe-coated-wire.html> |
| Thin aluminium metal mesh – window mosquito net | Leroy merlin | https://www.leroymerlin.pt/produtos/portas-janelas-e-escadas/redes-mosquiteiras/redes-mosquiteiras-para-janelas-e-portas/rede-mosquiteira-aluminio-1-2x2-5m-cinzento-12007163.html |
| Soldering Iron, Velleman VTSSC40N | Velleman | <https://www.velleman.eu/products/view/?id=353578> |
| Sandpaper 80g | - | - |
| Sterile Metal Screws, M1.2 | - | - |
| PTFE microminiature hook-up wire (n/a), 36744MHW | Phoenix Wire | <http://www.phoenixwireinc.com/MHW_TDQ_Specification_Chart.pdf> |
| Screwdriver, Xcelite R3322 | Xcelite | <https://www.amazon.com/Xcelite-R3322-Slotted-Screwdriver-Overall/dp/B07XYHB8PV> |
| ***Surgery*** | | |
| O2 medicinal | Linde Conoxia 100%, 50L, UN 1072 | <https://www.linde-gas.pt/shop/pt/pt-ig/gases-medicinais-gases-medicinais/ventila%C3%A7%C3%A3o-respira%C3%A7%C3%A3o-ventila%C3%A7%C3%A3o-respira%C3%A7%C3%A3o/conoxia-b20-%28un1072%29-2020123-103> |
| HIQ mano redutor | Linde | <https://www.linde-gas.pt/pt/products_and_supply/equipment/equipment_specialty_gases_hiq_/index.html> |
| EZ anesthesia | EZ Systems Corporation, EZ-108SA | <https://www.ezsystemsinc.com/product-category/anesthesia-machines-vaporizers/> |
| Magnifying glass Zeiss, Stemi 508, | Zeiss | <https://www.microscopeworld.com/p-940-zeiss-stemi-508-engraving-jewelry-design-microscope.aspx> |
| ZEISS Cold Light Source CL 6000 LED | Zeiss | <https://www.fishersci.se/shop/products/cold-light-source-5/11997281> |
| Nanoliter 2010 Microinjection Pump | World Precision Instruments | <https://www.wpi-europe.com/products/pumps-and-microinjection/oocyte-injection/nanoliter2010---discontinued.aspx> |
| Stereotaxic frame | Stoelting | <https://stoeltingco.com/Neuroscience/Just-for-Mouse-Stereotaxic-Instruments~9640?navigate_from_document=958&navigated_from_object=3776> |
| ,Standard Manipulator Arm | World Precision Instruments | <https://www.wpiinc.com/var-505214-standard-manipulator-arms> |
| Heating Pad | Dreamland thermo therapy | https://www.amazon.co.uk/Dreamland-16052-shoulder-intelliheat-technology/dp/B017KTUSCI |
| Hand drill ESCORT III H20 | ESCORT | <https://www.dentaltix.com/pt/marathon/micromotor-escort-iii-com-pm-e-pedal> |
| Drill, Carbide Bur | Edenta, C1.104.010 | <https://www.edenta.com/en/tungsten-carbide-instruments> |
| Anesthesia Gas filter, Braintree scientific | Fisher | https://www.fishersci.com/shop/products/f-air-cs-8-cs/NC9728504 |
| Isoflurane | Isoflurin 1000 mg/g | - |
| Povidone-Iodine 10% topical solution | - | - |
| Ethanol 70% | - | - |
| Gentian Violet solution | - | - |
| Buprenorphine  Bupaq 0.3mg/mL | Richter Pharma | 0.05mg/kg, subcutaneous  SC injection  dilution in 0.9% NaCl |
| Meloxicam  Meloxidyl 5 mg/mL | Ceva | 1 mg/kg, intraperitoneal  IP injection  dilution in 0.9% NaCl |
| Ophthalmic ointment Clorocil, eye ointment with chloramphenicol 10 mg/g | Clorocil®, Laboratório Edol | <https://edol.pt/produto/clorocil-10mg/> |
| Parafilm “M”, Bemis | Fisher | https://www.fishersci.com/shop/products/parafilm-m-laboratory-wrapping-film-2/1337410 |
| PBS 1X | - | - |
| Insulin Syringes, 29G | Microfine | - |
| Octocolagen | Clarben laboratories | https://www.dentaleader.com/esponjas-octocolagen-40-un-c-005 |
| Cotton Swabs | - | - |
| Pipette Tips | - | - |
| LY-A180 Wireless Led Dental Classic Curing Light Lamp |  | https://www.ebay.com/itm/266124664845 |
| Dental Cement Relyx Unicem 2 Automix, 3M A805-0592 | 3M | <https://www.3m.com.pt/3M/pt_PT/p/d/v000096373/> |
| Liquid Vaseline | HIGIUM |  |
| Nitrile Gloves | - | - |
| Hemostats 13003-10 | FST | <https://www.finescience.com/en-US/Products/Forceps-Hemostats/Hemostats/Hartman-Hemostats/13003-10> |
| Forceps, 11052-10, 0.6mm | FST | <https://www.finescience.com/en-US/Products/Forceps-Hemostats/Standard-Forceps/Graefe-Forceps/11052-10> |
| Fine Scissor, ZFV003 #1SF | Zeffiro Lascod | <https://www.dlcexpress.com/products/gum-scissors-zeffiro-italy> |
| Glass Pipettes for Nanoliter 2011, 504950 | World Precision Instruments | <https://www.wpiinc.com/504950-glass-capillaries-for-nanoliter-2010-fire-polished> |
| Scale, Compact Series, CSC 201 | Fisher Scientific |  |
| 1,1′-Dioctadecyl-3,3,3′,3′-tetramethylindocarbocyanine perchlorate | Sigma | https://www.sigmaaldrich.com/PT/en/product/sigma/42364 |
| Ringer Solution, Braun Vet | B. Braun Vet Care | - |
| Silk suture DS15 50 5/0 | Seraflex | <https://www.serag-wiessner.de/en/products/non-absorbable-suture-material/seraflex-silk> |
| Minocycline | 2mg/mL,13614-98-7 Acros |  |
| ***Silicon probe recovery and reuse*** | | |
| 1% tergazyme | Alconox | https://www.alconox.com/product/tergazyme/ |
| ***Histological confirmation of the recording sites*** | | |
| 1X phosphate buffered saline (PBS) | - | - |
| 4% paraformaldehyde (PFA) | Acros | - |
| NGS | Sigma | - |
| Triton | Acros | - |
| Anti Iba1, Rabbit (for Immunocytochemistry) | FujiFilm Wako | - |
| Vectashield (VECTASHIELD HardSet Mounting Medium with DAPI | Vector | - |

**Table 3.** Troubleshooting.

| **Problem** | **Possible causes** | **Solutions and tips** |
| --- | --- | --- |
| Recovery from anesthesia is too prolonged | Surgery time is too long, anesthesia potency is too high. | - Train and optimize all steps in cadavers: with practice, the surgery time will decrease.  - Perform mock surgeries first without silicon probes to practice placement of the nanodrive(s) and dental cement application. Employ surgery with silicon probes only after all previous steps are well optimized.  - Optimize anesthesia route concentration. We recommend inhaled anesthetics as the recovery can improve. Inhaled anesthetics also provide a finer control over depth of anesthesia. |
|  | Excessive blood loss during surgery | - Control bleeding as soon as it happens using hemostatic sponges.  - Adjust craniotomy localization as some brain regions are more irrigated than others.  - Administer saline during and after surgery to prevent fluid loss. |
|  | Temperature decreased drastically | - Monitor temperature throughout the procedure and use a heating pad accordingly. However, if using heating elements, consider administering a greater amount of fluids. |
| Prolonged post-surgery recovery | Multiple | - Use the mouse grimace scale as a guide to monitor for any signs of pain and administer analgesics accordingly.  - Use an antibiotic (e.g. minocycline) to help with recovery. Minocycline have the added benefit of delaying gliosis around the silicon probe.  - Monitor weight, water and food consumption. Apply wet chow with peanut butter or other appetitive, high caloric food, for 3-4 days to prevent excessive weight loss and accelerate recovery.  - Consider administering saline daily via IP injections. |
| Can I target other brain regions? |  | - Use the 3D models described in this paper to plan the best placement for your nanodrive(s) and base placement for your target brain region(s):  - If the target brain regions are very close, consider a single craniotomy (that does not exceed 1 mm in diameter) and the use of silicon probes with pronged or forked shanks.  - For more distant brain regions, change placement architecture accordingly.  - If placement proves to be difficult for your target brain region(s), consider modifying the shape of the base and lateral walls to suit your needs (e.g., increasing the base’s width so it has more room to accommodate the nanodrive(s)). |
| Headgear does not feel sturdy after placement | Base is not adjusted perfectly with the skull. | - To prevent the base from detaching, ensure that dental cement is placed all around the outer ridge of the base, securing it to the skull and supporting screws.  - The base must be positioned in such a way that allows for the placement of the nanodrive(s) in your desired coordinates, while still maintaining a tight fit alongside the skull. This can lead to some parts of the base not being in direct contact with the skull. If there is a visible gap between the skull and the outside part of the base, apply dental cement to further solidify the structure from inside and outside.  - Do not place cement in the muscle or skin area.  - In the final stages of the surgery, after placing the reference and ground wires in contact with the cerebral spinal fluid, apply more dental cement in this craniotomy, joining the base, the skull, the screws and the wires in a single structure. |
|  | Fitting not correct between the base and lateral walls | - The lateral walls should couple with the base without difficulty and be sturdy enough on their own. If this connection is not secure, add dental cement in the inside and outside connections between the base and lateral walls. |
|  | Support screws not properly attached to skull | - After placement of the support screws in the skull, confirm they are properly attached by slightly nudging them and see if the skull moves along. If not, and without damage to the cortex, apply additional turns until the screw is fully stable.  - The hole bored for the screw should be slightly smaller than the screw diameter.  - If using self-tapping screws, grind away the pointy tip as it may damage the cortex and prevent a tighter fit. |
|  | 3D printed pieces are not well cleaned | - Depending on the printing process, some 3D pieces may still have residues that prevent a tight fit between the components of the headgear. Make sure all the connection areas are free of any accumulated residue and test the assembled headgear before surgery |
| Silicon probe bending while lowering | Craniotomy too dry | - Apply small drops of PBS or paraffin directly to the craniotomy before lowering the nanodrive. |
| Encasement stability | Printing quality and/or material used | If the encasement for the nanodrive and the detachable base is not secure, this could lead to unwanted drift in your recordings as the silicon probe may move with the encasement.  - The selection of the printing material and printing process is critical and should be optimized to ensure easy recoverability. Even minor changes will compromise the stability of the encasement and detachable base. Testing different materials (or even the same material in different colors), can have a dramatic effect in the final outcome.  - As a final solution, dental cement can be added between the encasement and the detachable base. This will compromise removability of probes. |
| Noise in the recordings |  | - Line noise (50Hz or 60Hz) contamination in your recordings may be due to the reference and ground wires not being properly in contact with the cerebral fluid. Make sure that the wires are properly placed and secured inside the craniotomy.  - Make sure all your equipment is properly grounded and test light sources as a potential contaminant. |
